# Competence perceptions of veterinary nursing students and registered veterinary nurses in Ireland: a mixed methods explanatory study

**DOI:** 10.1186/s13620-020-00162-2

**Published:** 2020-06-17

**Authors:** Karen Dunne, Bernadette Brereton, Vivienne Duggan, Deirdre P. Campion

**Affiliations:** 1 Department of Agriculture, Food and Animal Health, Dundalk Institute of Technology, Dublin Road, Dundalk, Ireland; 2 Centre for Excellence in Learning and Teaching, Dundalk Institute of Technology, Dublin Road, Dundalk, Ireland; 3 UCD School of Veterinary Medicine, University College Dublin, Belfield, Dublin 4, Ireland

**Keywords:** Veterinary nurse, Competence, Day-one competencies, Competency, Workplace, Experience, Confidence

## Abstract

**Background:**

Veterinary regulators require veterinary nursing students to demonstrate clinical competence prior to registration and practice as a veterinary nurse. However, in common with other medical professions, there is no one broadly accepted definition of competence. Studies in nursing have revealed that practicing nurses may view newly qualified colleagues as lacking competence, leading to disillusionment with nursing training programmes. Similar studies are lacking in veterinary nursing, despite the profession having recently undergone a similar transition from workplace-based training to undergraduate education.

**Methods:**

A mixed methods explanatory study surveyed 66 Irish registered veterinary nurses and 31 first year veterinary nursing students at two Irish third level institutions to obtain their views on what constitutes veterinary nursing competence and when veterinary nurses develop it. The surveys were followed by student focus groups and semi-structured one-on-one interviews with registered veterinary nurses. Content analysis was employed to analyse the surveys, while the focus groups and interview transcripts underwent thematic analysis.

**Results:**

Students perceived competence primarily as the ability to provide patient care, and they expected it to develop close to the time of graduation. RVNs held a broader definition of competence, incorporating leadership skills and confidence as well as patient care provision. RVNs expected it to take approximately two years of workplace-based experience post-graduation for a veterinary nurse to develop competence. In addition, RVNs recognised that anxiety felt by many newly qualified veterinary nurses during this period could be attenuated by mentorship from more experienced colleagues.

**Conclusions:**

Irish RVNs and veterinary nursing students perceive competence differently, similar to previous findings from the nursing profession. Educators and regulators should provide explicit descriptions of terms such as ‘competence’ to avoid confusion and possible disillusionment amongst veterinary nursing stakeholders.

## Introduction

### Veterinary nursing competence

The Accreditation Committee for Veterinary Nurse Education (ACOVENE) defines competence as “a cluster of related skills, knowledge and attitudes that enable an individual to carry out a function in a range of situations and/or contexts” [1] p.7. Similarly, the Royal College of Veterinary Surgeons (RCVS) defines veterinary nursing competence as “the ability to perform the roles and tasks required of one’s job to the expected standard” [2] p.1. Both these regulatory bodies recognise that competence is a relative term, as increasing levels of performance will be required throughout a career. Therefore, they specify ‘Day One’ competencies as being at the level and range expected of a newly qualified veterinary nurse.

### Definitions of medical competence

The term ‘competence’ does not have a single, broadly accepted and widely understood meaning. Competence is defined in the Collins English Dictionary as ‘the condition of being capable’ or having ‘ability’, with competency being noted as an alternative and less commonly used form of competence [3]. Competent refers to ‘having sufficient skills or knowledge’ or ‘being suitable for purpose’ [3]. Given the interchangeable nature of ‘competence’ and ‘competency’ in the English language, there is a degree of overlap in the medical literature relating to these terms, with authors either using them interchangeably or noting that they may be so used [2, 4–9]. Indeed Fernandez et al. remark that:

a straightforward examination of what is meant by the term ‘competence’ is noticeably missing from the literature, despite its impact on medical training [10] p.357.

Two recent reviews of medical competence [6, 11] agree that ‘competency’ should be reserved to describe an individual skill or task which is an “observable ability of a health professional” [6] p.641. ‘Competent’ and ‘competence’ refer to the performance of a person, while ‘competency’ relates to skills. ‘Competence’ is the ability to perform a skill and is therefore an attribute possessed by a ‘competent’ person, while competencies are the tasks that must be performed in a person’s role or job. Individual competencies may be combined into a ‘competency framework’, which identifies the skills or abilities a learner must achieve or demonstrate to be deemed competent in a particular discipline [5, 8, 9, 12]. Table 1 defines these terms as they are used in this article.

**Table 1:** Definitions related to competence and competency

Competence	Ability to perform a skill; a personal attribute.
Competent	An adjective used to describe a person’s performance that is deemed acceptable or to an expected level.
Competency	An individual skill or task which must be performed as part of a person’s job or role (plural = competencies).
Competency Framework	A set of skills a learner must achieve or demonstrate to be deemed competent in a particular discipline.

### Competence as a performance standard

The term ‘competence’ is also widely used in the literature to refer to a point on a scale of performance ability from beginner/novice through competence/proficiency to expertise or mastery [11, 13–17]. Competence is therefore a stage in the learning process, not the final destination. However, a learner may need to attain competence on such a performance scale to achieve certain high-stakes educational benchmarks e.g. be deemed ready to graduate [16].

**Fig. 1:**
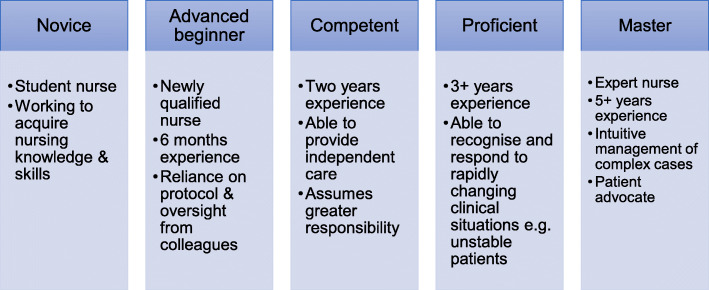
The stages of the Dreyfus Model of Skill Acquisition as applied to nursing education by Benner et al. [19]

Patricia Benner applied the Dreyfus Model of Skill Acquisition to the development of nursing expertise [14, 18]. The Dreyfus Model classifies the stages of learning from novice to mastery via three intermediate stages, namely advanced beginner, competent and proficient (Fig. 1) [13]. Benner and her co-authors proposed that as a trainee nurse gained experience and progressed through these stages, their performance shifted from a reliance on information and rules to an intuitive appraisal of complex clinical situations [19]. At least five years’ workplace experience was considered necessary by these authors to attain nursing expertise. Newly qualified nurses (with approximately six months’ work experience) were viewed as advanced beginners by Benner [19]. Deliberate practise refers to the focused repetition of a skill with the goal of improved performance and is necessary for learners to progress through competence to expertise [20, 21]. 10,000 h, or ten years of daily practise, has been suggested as the timeframe necessary to achieve mastery [22, 23].

Benner and her colleagues also recognised that this progression from beginner to expert elicited an emotional response in learners. Newly qualified nurses reported being anxious, as they recognised that they were now required to assume responsibility for patient care without yet feeling fully confident in their ability to do so: “I’m not the student just watching and observing. I have to actually do something” [19] p.19. This anxiety could be at least partly ameliorated by support from more experienced colleagues.

### ‘Day one’ versus ‘professional’ competence

**Fig. 2:**
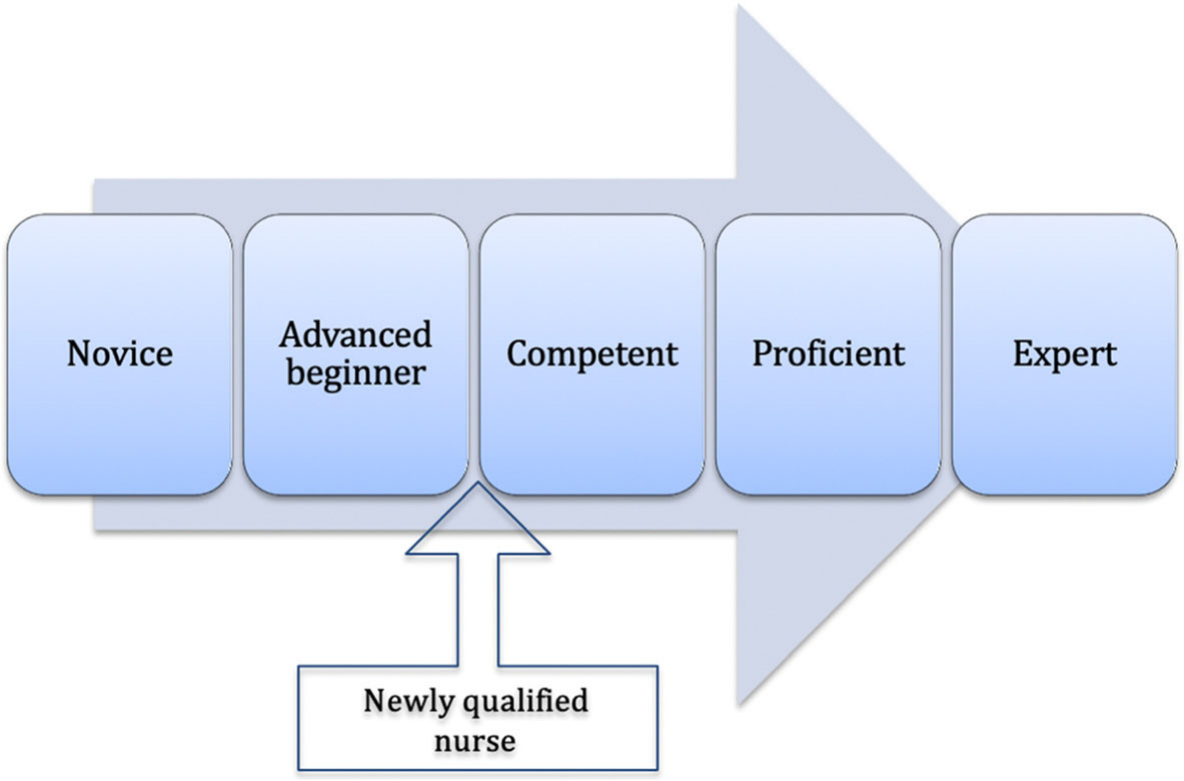
Dreyfus and Dreyfus five-stage model of competence (1980) with Benner’s (1984) newly qualified nurse as an advanced beginner

‘Day one’ competence therefore corresponds to the level of advanced beginner on Benner’s framework, as learners at both these stages require additional experience to become competent: meaning, able to practice independently (Fig. 2). The Irish and UK veterinary regulators recognise this by acknowledging that newly qualified veterinary professionals require additional guidance from their employers and more experienced colleagues as they begin to practice their profession [2, 24, 25]. Oversight and support from colleagues is required to mitigate anxiety and help new graduates develop the confidence and capabilities needed to practise safely [24–26].

‘Professional competence’ has been suggested as a term to distinguish those who have gained clinical experience in the practice of their profession from newly qualified veterinary nurses [27]. These distinctions amongst levels of competence may cause confusion: a ‘day one competent’ person is unlikely to perform at the same level as a more experienced ‘professionally competent’ colleague.

The definition of competence and the identification of ‘day one competencies’ are highly relevant to veterinary nursing educators, as these are the criteria on which curriculum design and teaching practices are based. However, Irish veterinary nursing students also spend time on work placement in veterinary practices during their training, typically alongside registered veterinary nurses (RVNs). Work placement colleagues and supervisors, therefore, have an important (albeit mostly informal) role to play in the training of veterinary nursing graduates. It is critical that members of the profession who are working with and supervising students in the workplace are aware of the standard that students are trying to attain before graduation. The potential for confusion exists if educators and practicing professionals perceive competence differently, as students may find different expectations placed upon them.

In addition, if practicing nurses perceive competence in a different way to educators, they may find students and new graduates to be ‘unable’ or not yet competent, leading to disillusionment with the standard of education being provided [4, 28–30]. The nursing literature contains reports that experienced nurses may consider newly qualified colleagues to be incompetent or not ‘work-ready’ [4, 31]. Such feelings could result in disillusionment and has led to criticism of nursing training programmes [31]. The transition from the ward to the lecture theatre that nursing education has undergone could be exacerbating this, with experienced nurses potentially feeling that modern training is primarily focusing on theory, to the detriment of practical skills acquisition [29, 32]. The veterinary nursing profession has recently undergone a similar shift in training; away from apprentice-type, practice-centred training to an undergraduate, campus-based model [33, 34]. There is a lack of research examining the effect of this transition on the competence perceptions of practicing veterinary nurses and their views on the workplace readiness of newly qualified members of their profession.

An Australian study of almost 300 experienced nurses found that the majority of them considered newly qualified nurses to be unable to practice independently in many areas [30]. Possible reasons for this include the increasing complexity of nursing care provision and the transfer of nursing training from hospitals to third level institutions, with a resulting decrease in the time spent gaining ‘hands on’ clinical experience in the workplace [29]. Other authors have examined the perceptions of nursing students and recent graduates of their readiness to practice and found a lack of confidence and feelings of incompetence to be common [4, 35, 36]. However, there are no reports relating to the competence perceptions of veterinary nursing students or RVNs. It is this literature gap that this study was intended to address.

### Study aims

The purpose of this exploratory study was to explore how Irish RVNs and veterinary nursing students view competence and how and when they expect it to develop. Three research questions (RQs) were identified to achieve these aims:

RQ1 What do veterinary nursing students and RVNs expect a competent veterinary nurse to be able to do?

RQ2 When do they expect a veterinary nurse to become competent?

RQ3 What is the role of experience in the development of competence?

## Methodology

### Study design

**Fig. 3:**
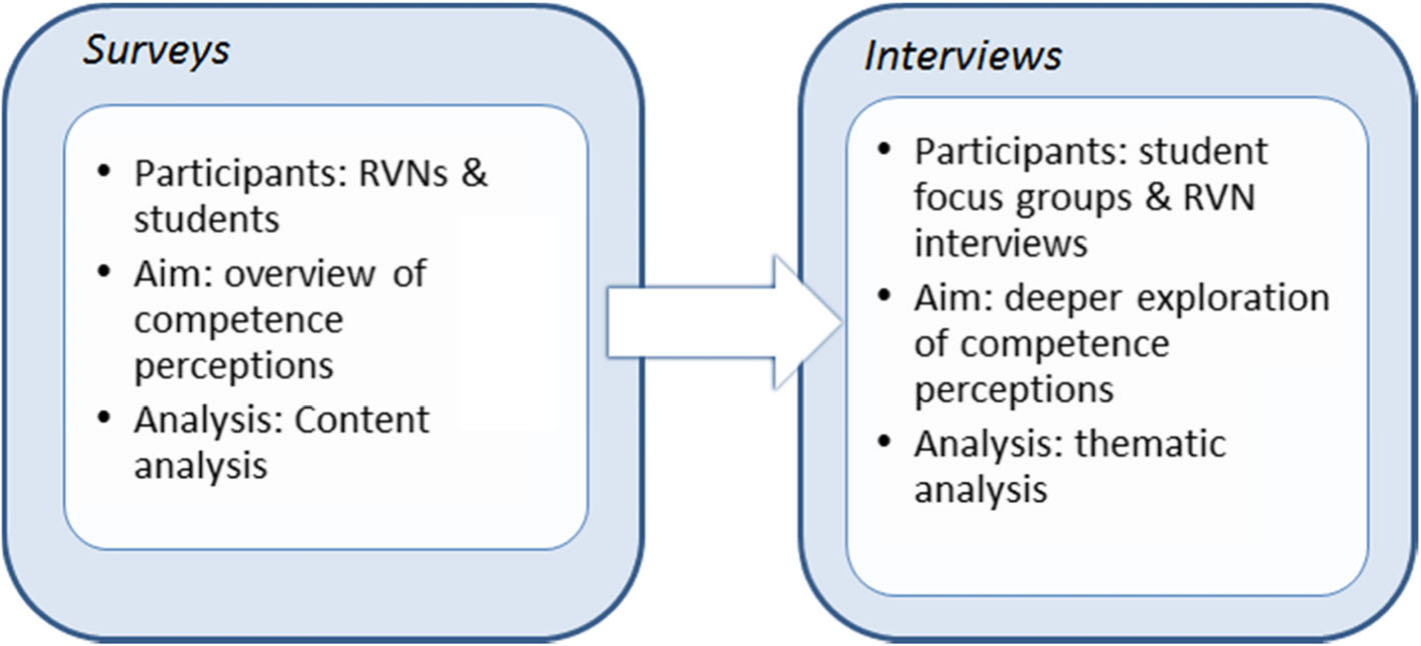
Explanatory sequential study design overview

A mixed methods explanatory sequential design of surveys followed by interviews was selected [37]. While an exploration of competence perceptions lends itself to qualitative enquiry, the many interpretations of the term and the lack of pre-existing literature on the views of veterinary nurses justified an initial phase consisting of cross-sectional group comparison surveys (Fig. 3). These surveys were intended to obtain a broad view of competence perceptions across RVNs and veterinary nursing students. The surveys were followed by qualitative interviews with RVNs who had experience of supervising students during work placement and student focus groups to obtain deeper insights into the views and beliefs expressed in the survey responses. This process facilitated triangulation of the survey and interview data during analysis. The aim was to enhance the validity of the study via mutual corroboration [38].

### Survey data collection

RVN and student web-based questionnaires were distributed using SurveyMonkey. The questionnaires included some demographic information: year of birth and gender. Additional data collected from RVNs consisted of year of graduation, qualification obtained and current area of work (Appendix 1).

With regards to competence, an open-ended item asked respondents what the term “competent” meant to them (RQ1). Closed items asked respondents to indicate when they felt a veterinary nurse became competent versus experienced (RQs 2 and 3). The survey was piloted with a number of veterinary nurses and students to check for question clarity prior to data collection.

### Participants

A typical sampling strategy was employed [39]. All first year veterinary nursing students at Dundalk Institute of Technology (DkIT) and University College Dublin (UCD) aged 18 years or older were invited to participate in the study. The survey link was emailed to the students following a face-to-face briefing session with the author to explain the project. The survey details and an invitation for RVNs to participate were emailed to the 81 registered veterinary premises on the DkIT veterinary practice work placement database. The practices were asked to inform their RVN employees about the project. The Irish Veterinary Nursing Association (IVNA) also emailed the survey invitation to their membership, which numbered 472 individuals at the time of the survey (December 2014).

### Survey analysis

The closed-ended survey items were exported into Microsoft Excel and examined using descriptive statistics. The open-ended statements underwent content analysis using a quantitative research approach which assesses word frequencies [40] and has been used in nursing research to study participants’ experiences without loss of context [41, 42]. The responses were collated, read through repeatedly and first divided into ‘meaning units’ [43]. These were further condensed into codes, counted and grouped into categories following multiple passes through the data. Finally, the categories were abstracted into themes that expressed the latent meaning of the responses. The counting of the codes is an aspect of quantitative content analysis that enables the researcher to identify aspects of the research phenomenon that are frequently referred to by participants [44]. This allows the data to be presented in a manner that is representative of the population sampled [41].

The findings from these survey items were used to review the focus group and interview questions. The survey results indicated a difference between students and RVNs in the contribution of experience to competence development. The students, in general, expected to become competent close to the time of graduation and then add additional clinical experience. RVNs, in contrast, saw workplace experience as a prerequisite; which resulted in an expectation that a veterinary nurse would require 2–3 years of workplace experience after graduation to be considered competent. These different views of the contribution of clinical experience to competence attainment between the two survey groups led to an additional interview question: “what is the role of experience in becoming competent?” (RQ3).

### Interview data collection

Two student focus group interviews were held, in DkIT and UCD respectively. Group interviews were conducted with the student participants both for practical reasons of efficiency and minimising timetable disruption [45] and to reduce the power differential between undergraduate students and academic researchers [46, 47]. All students who had completed the survey were invited to take part. Six DkIT students (five females, one male) and eight UCD students (all female) volunteered to take part in the focus groups. Each session lasted approximately one hour and was moderated by the second author. The first author was also in attendance to moderate any veterinary-specific queries that arose but did not contribute to the discussion. See Table 2 for the focus group questions.

**Table 2:** Student focus group questions

You are expected to become a competent veterinary nurse. What does that term “competent” mean to you? When would you expect a veterinary nurse to become competent? What is the role of experience in becoming competent?	

The DkIT veterinary practice work placement database was used to identify registered veterinary nurses employed by veterinary practices in the Republic of Ireland who had previously acted as a student work placement supervisor and were at least three years qualified (as the survey data showed the majority of respondents indicated that three years of experience after graduation was the time needed to be competent). RVNs with experience of student supervision were invited for interview to ensure that they had insights in the development of competence both during veterinary nursing training and after graduation. 33 RVNs met these criteria and were invited, via an email outlining the purpose of the study, to volunteer to be interviewed. Individual interviews were conducted with these participants due to the logistical challenges associated with organising a group meeting of RVNs working in private practices across a large geographical area, and the absence of a power-over relationship between these volunteers and the researchers [45, 48].

Of the 33 RVNs invited for interview, seven initially agreed to take part. One volunteer was subsequently unavailable due to work commitments. Of the six RVN interviewees; all were female, two worked wholly with small animals, two worked in specialist equine practice and two in mixed practices (Table 3). They had all obtained their veterinary nursing qualification in Ireland and their duration of experience in practice ranged from 4 to 13 years (M = 7.83, SD = 3.71).

**Table 3:** Registered veterinary nurse interviewees

Interviewee	Current employment	Gender
RVN1	Mixed practice	Female
RVN2	Equine practice	Female
RVN3	Equine practice	Female
RVN4	Mixed practice	Female
RVN5	Small animal practice	Female
RVN6	Small animal practice	Female

A one-on-one semi-structured interview format was used to maintain consistency in how the interviews were conducted while allowing participants to elaborate on their views. The open-ended interview questions (Table 4) were followed with probes and checks to explore answers and confirm accuracy of interpretation [49]. All interviews were conducted by the first author at a location and time convenient for the interviewee.

**Table 4:** RVN interview questions

Picture a competent veterinary nurse in your mind. Could you please describe that person to me? When does a veterinary nurse become competent? What is the role of experience in becoming competent?	

### Interview analysis

The focus groups and one-on-one interviews were audio-recorded and transcribed by the first author using the Google Docs Voice Typing dictation software tool [50]. The transcripts were anonymised and then shared with the focus group and interview participants to check their veracity prior to analysis. Thematic analysis [51] was performed on these transcripts by the first author, with the second author acting as an independent second coder. Following several readings to become familiar with the content, the transcripts were inductively open-coded for relevant themes using the spreadsheet method described by Bree and Gallagher [52] (Table 5). The codes were then reviewed by both coders to remove overlaps and consolidated by consensus into themes that reflected the participants’ views on competence.

**Table 5:** Qualitative data analysis overview

Data analysis steps performed	Thematic analysis phase (Braun & Clarke, 2006)
Focus groups and interviews were audio recorded	
Discussions transcribed in Google Docs, anonymised and transferred to Microsoft Excel	1
Transcripts verified by participants to confirm their accuracy	1 & 2
Initial read through the data centred on the identification of themes	2 & 3
Themes were initially coded by cell colour to match thematic areas	3, 4 & 5
Microsoft Excel’s filter applied to sort the data by cell colour (grouping codes into thematic areas)	3, 4 & 5
Second pass over data identified overlaps and consolidated data points	4 & 5
Numerous further passes over data, condensing data at each stage to collapse codes into themes	5 & 6
Generation of data overview summary with key points under the three main themes which emerged	6

## Results

### Survey results

#### Demographics

Thirty-one students (14 from DkIT and 17 from UCD) of the total eligible cohort of 74 completed the survey for a response rate of 42%. 9.7% of the student respondents were male (*N* = 3) and 91.7% (*N* = 28) were female. Their average age was 23 (±7) years.

Sixty-six completed survey responses were received from RVNs (65 females, 1 male). The IVNA had 472 members at the time of the survey, in late 2014, so this represents an approximate response rate of 14%. It also reflects the gender balance (95% female, 5% male) of the Irish veterinary nursing profession [53].

The majority (85%, *N* = 56) of the veterinary nurses who responded to the survey held an Irish veterinary nursing qualification with the remainder holding a RCVS diploma from the UK (15%, *N* = 10). The RVN respondents had been qualified for seven (±7.6) years on average and had a mean age of 34 (± 9) years.

**Fig. 4:**
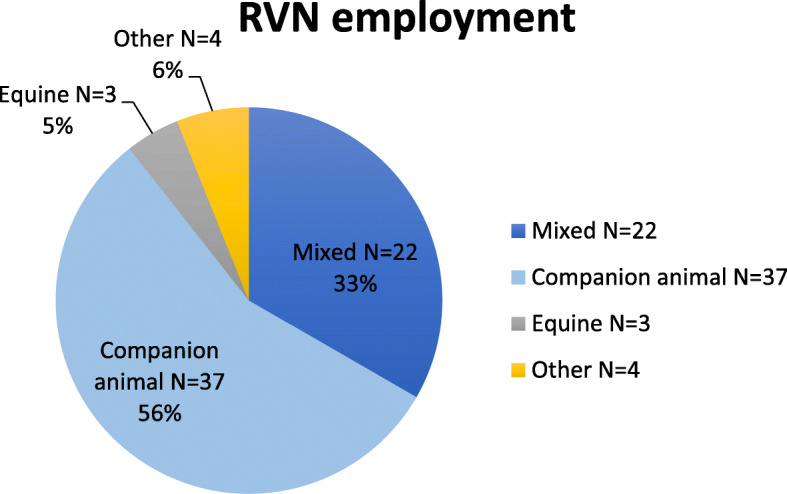
Areas of employment of RVN respondents

Just over half of the RVNs (56%, *N* = 37) were working in companion animal practice with mixed and equine practice employment reported by the remainder (Fig. 4). ‘Other’ employment roles consisted of practice management, an animal charity, industry sales, and education.

#### Time taken to become competent and experienced:

**Fig. 5:**
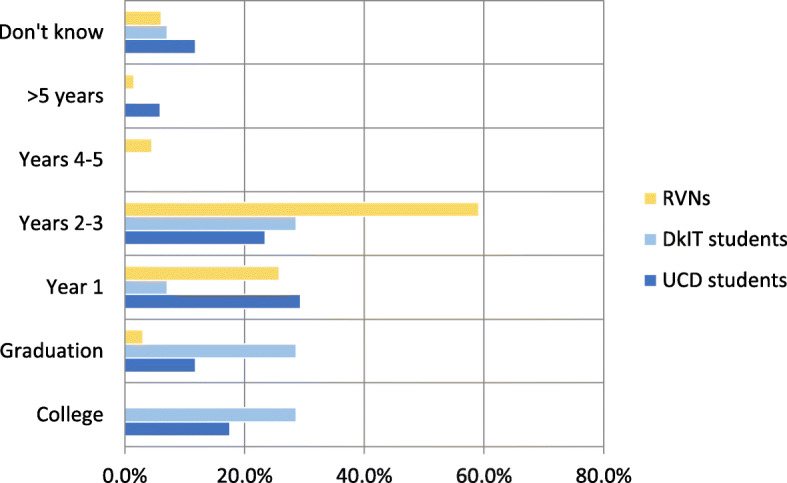
Time taken for a veterinary nurse to become competent

Thirty-one students (14 from DkIT and 17 from UCD) and 66 RVNs completed this section of the survey. Of these, 42% (*N* = 13) of students expected a veterinary nurse to become competent during college or by graduation, but only 3% (*N* = 2) of RVNs shared this view (Fig. 5). The majority of RVNs (84.9%, *N* = 56) expected a veterinary nurse to be competent within three years of graduation. This view was shared by 45.2% (*N* = 14) of the students. There was no correlation found between the original training route completed by the RVN and their expectation regarding time to achieve competence.

**Fig. 6:**
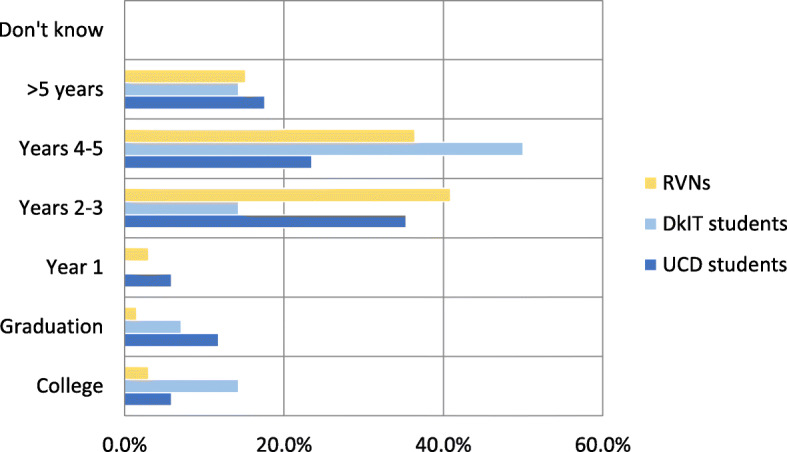
Time taken for a veterinary nurse to become experienced

Most respondents expected a veterinary nurse to need several years of workplace exposure post-graduation to develop experience (Fig. 6). Only 22.6% of students (*N* = 7) and 7.5% of RVNs (*N* = 5) expected experience to be attained during training or within the first year of a career. 61.3% of the students (*N* = 19) and 77.3% of RVNs (*N* = 51) felt it would take 2–5 years in the workplace to achieve and the remaining five students and ten RVNs (16.1 and 15.2% respectively) expected more than five years to be required. Overall, students tended to expect experience to follow competence while RVNs, in contrast, felt the development of these two attributes overlapped.

#### The meaning of ‘competent’

Respondents identified numerous features of a competent RVN, grouped into four main themes, namely knowledge, performance, professional behaviour and confidence. While both students and RVNs identified all these themes, the two groups of respondents differed in the emphasis they placed on the thematic constituents. This difference was identified by quantifying the number of times respondents referred to the codes that constituted the final themes (Table 6).

**Table 6:** Content analysis of survey competence statements: frequency of codes and themes identified by students and RVNs

Theme	Constituent codes	References by students % N		References by RVNs % N	
Knowledge	Knowing	33	9	67	18
	Understanding	14	1	86	6
	Apply knowledge	36	5	64	9
Performance	Able to do	33	27	67	55
	Skilful	50	5	50	5
	To a standard	27	3	73	8
	Safe	17	1	83	5
	Experience	17	1	83	5
	Without error	0	0	100	2
Professional behaviour	Efficient	33	2	67	4
	Trustworthy	50	2	50	2
	Work without help	20	1	80	4
	Ask for help	50	1	50	1
	Being responsible	0	0	100	3
	Qualified person	0	0	100	4
	Use initiative	0	0	100	5
	Teach others	0	0	100	1
	Leadership	0	0	100	2
Confidence	Confident	17	4	83	19

#### Knowledge theme

Both students and RVNs referred to the need for a competent person to possess the theory necessary to practice veterinary nursing, taking account of risk appraisal and subsequent decision-making.

#### Performance theme

All respondents emphasised the ability of a competent person to carry out clinical skills and practical tasks: “ability to do the job” (RVN 66). The RVN responses more commonly referred to additional aspects of job performance by a competent person, such as being able to complete tasks safely and efficiently without error, assistance or supervision. Five RVNs, but only one student, referred to ‘experience’ as a necessary component of competent performance.

#### Professional behaviour theme

Being able to work efficiently and without assistance but also trusted to recognise when to ask for help were expected behaviours by both students and RVNs. In addition, RVNs expected a competent person to be “able to work on one’s own initiative” (RVN 15) and “take [on] responsibility within the workplace” (RVN 44). Leadership, teamwork and the ability to teach others were additional traits of a competent individual identified by RVNs.

#### Confidence theme

19 RVNs and four students referred to ‘confidence’ in relation to competent performance. Self-confidence and an ability to do the job without “second-guessing yourself” (RVN 69) was seen as relevant by RVNs, in addition to confidence when interacting with colleagues and dealing with clients

### Focus group and interview results

#### Students and RVN perceptions of a competent veterinary nurse:

The themes that emerged from the interviews mirrored the survey findings: competent veterinary nursing practice requires integration of theoretical knowledge, clinical performance and professional behaviours. Reflective practice was identified as another necessary attribute while confidence and context emerged as influencing factors (Table 7).

**Table 7:** Focus group and interview attributes of a competent veterinary nurse

Themes	Subthemes	Students	RVNs
Attributes	Knowledge	Theoretical knowledge.	Theoretical knowledge.
	Performance	Practical patient care clinical skills. Work independently.	Practical patient care clinical skills. Work independently. Provide holistic patient care (primary responsibility). Plan ahead, work efficiently. Use initiative.
	Professional behaviour	Adaptable. People skills. Responsible. Manage emotions. Trustworthy. Recognise limits and ask for help.	Adaptable. People skills. Responsible. Manage emotions. Trustworthy. Recognise limits and ask for help. Support colleagues. Leadership. Admit mistakes. Love of the job.
	Reflective practice	Keep learning.	Keep learning. Constant re-evaluation of patients/situations. Self-appraisal.
Confidence		Confidence	Confidence
Context		Competence varies with species.	Competence varies with species. Caseload affects competence development.

RVNs had a broader and more nuanced view of competent performance and identified more constituent components of it than students. This wider view of competence held by RVNs is not a surprising finding due to the extensive clinical experience of the RVN interviewees.

#### Attributes of a competent veterinary nurse

Both students and RVNs expected a competent individual to possess knowledge of relevant theory and be capable of applying it to patient care: “it’s as important to be a practical person as it is to have the knowledge behind you” (RVN 4). Participants also emphasised the need for a competent veterinary nurse to be able to perform independently in the clinical workplace (with a focus on patient care).

RVNs also emphasised the need for a competent veterinary nurse to be able to work efficiently and use their initiative to prioritise tasks. “[A competent veterinary nurse]; they’re always looking ahead, they’re always trying to pre-empt what is going to happen next. Rather than just dealing with what is happening at the time” (RVN 5).

A range of intra- and inter-personal skills, such as being responsible, adaptable, and good at dealing with people were highlighted, as well as intrinsic motivation of the love for the job: “that’s the most important thing: you have to love the job you’re doing” (RVN 1). Both students and RVNs felt that being trustworthy was important. They expected a competent person to possess the self-awareness to recognise when they needed help and to ask for assistance. “Part of being competent is to admit that you’re not in a position to be able to deal with everything … You have to know your limits” (DkIT student).

In addition, RVNs emphasised the need to be able to admit mistakes and not only support colleagues but also direct or manage the practice team when necessary.

Even when things might have gone the wrong way and people are upset or whatever, you need to manage that … You know, be able to point out where the mistake was so it doesn't happen again. But also point out that these things do happen and it's not the end of the world (RVN 5).

Both students and RVNs were aware that working as a veterinary nurse could exert an emotional toll, particularly given the potential for mistakes to lead to poorer patient outcomes. They felt that a competent person also needed the resilience to manage this burden.

And it can be very hard to come back after making a mistake. It's very, very hard because it is so disheartening. It's soul destroying do you know, especially if it was a mistake that didn't have to happen. You know we're all human, we will all make mistakes but I think it's a matter of trying to understand that it's just the job you're in. You made a mistake and you'll never make it again (RVN 5).

Reflective practice was identified as an important subtheme underpinning competence. Participants were aware that ongoing learning was necessary to keep abreast of clinical care developments. RVNs stated that as well as keeping up with patient care advances, competent nurses needed to self-appraise their practice. “You have to think for yourself, was I ok in that situation? What did I do? What could I have done better? What did I not do quickly enough? What do I maybe need to ask a more senior person?” (RVN 3).

Confidence was identified as an enabling factor for the attainment of competence: “if you can build confidence then competence will come a lot quicker” (RVN 5). A lack of confidence was felt to hinder independent, self-regulated practice. “[A competent person] can take instruction and have the confidence to go off, you know, and work to a certain extent on their own [but also] know when to ask for help” (RVN 2).

Finally, all participants recognised that competent performance is context-specific, i.e. related to the species and clinical specialities veterinary nurses are familiar with, and these skills may regress with lack of use.

#### When does a veterinary nurse become competent?

The student focus group discussion was similar to the survey findings in that participants expected to become competent close to graduation from college: “I wouldn’t expect it on day one in a new job but I’d hope that after a few months that I’d feel competent then” (DkIT student 1).

The RVN interviewees, also in line with the survey findings, expected it to take between 18 months and three years to become competent post-graduation. Four of the six interviewees nominated two years in practice as the expected timeframe. “I think it takes at least two years [for you] to say that you’re a good, confident, competent nurse” (RVN 4).

#### The role of experience

Some insight into why these time frames differed was provided by the discussions around the role of workplace experience in becoming competent [RQ3]. The three themes identified recognised the effects of workplace experience on the recently graduated veterinary nurse, the role of the veterinary practice, and the RVN as a mentor during this phase of initial clinical experience.

#### Workplace experience and the newly qualified veterinary nurse

Both students and RVNs recognised that a new graduate’s personality and prior experience would influence their rate of progress.

The overall character of the person … does play a massive role in it … the ones who have worked … before they come to college … Or those from a farming background … or equine background. They can become competent more quickly (RVN 2).

Students expected that workplace experience would primarily improve clinical skills performance and confidence while RVNs felt that its contribution was strongly related to improved self-efficacy, including an awareness of one’s own limitations and a willingness to ask for help; once again possibly related to the RVNs greater awareness of the complexities of the job.

Newly qualified nurses were expected to make some mistakes but also to learn from them: “mistakes happen. Talk about them, learn from them and move on” (RVN 4). Learning to appraise information and prioritise tasks was seen by RVNs as a vital constituent of competent performance that required experience to develop.

Just trying to juggle everything and know how to prioritise … you can [finish college] with your skills and then you just come in [to the workplace] and you don't know where to start or how to deal with people and it can be very overwhelming (RVN 2).

RVNs recognised that while newly qualified veterinary nurses had plenty of theoretical knowledge, they needed opportunities to apply theory to clinical cases, evaluate the outcomes and grow in confidence.

#### The role of the workplace

RVNs recognised the transition to the workplace and the assumption of responsibility for patient care as a potentially stressful time.

I remember the first [emergency surgery] because … I got the phone call at 4:30 in the morning. And my heart was going … you just get nervous...and the worry. But the high afterwards, especially as the animal was ok. It was amazing you know. Then the next one that came in, you just feel ‘ok, I know what to do now’ (RVN 6).

A supportive workplace environment assisted in successfully managing this transition and led to an increase in confidence for the new graduate, as well as ensuring an ongoing supply of capable nursing staff. “[Starting work] has to happen in order for you to gain confidence and become competent. You have to see that ‘ok I did that on my own and I was fine. I did it well’ and that’s when you gain confidence” (RVN 3).

#### RVNs as mentors

The veterinary nursing students’ discussion recognised the desirability of oversight by more experienced colleagues during the transition from college to the workplace. “If you feel uncomfortable there’s someone you can go to straight away. You can go and ask them and they’ll willingly come to you. They’re not waiting for you to mess up” (UCD student). The RVNs felt that this responsibility was something that they could and should assume, with inherent rewards: “it’s nice to be able to pass on your little tips” (RVN 5). “I think that it’s up to the nurses who are in the practice to really take [newly qualified RVNs] under their wing and be their backup and kind of be there for them if anything does happen” (RVN 4).

Aspects of mentorship identified including: allowing new graduates to assume responsibility and become competent without being overwhelmed, and reassurance that they were not expected to be able to do everything immediately. “There will be a certain amount of hand holding but then the stabilisers come off and [they] are on [their] own” (RVN 3).

The RVN interviewees also referred to a tendency for newly qualified veterinary nurses to feel that they had to take on aspects of patient care that were beyond their remit, for example knowing which medications would be required in an emergency situation. These veterinary nurses recognised this tendency to assume too much responsibility as a source of anxiety for inexperienced colleagues.

I think [newly qualified] nurses get themselves very flustered as to ‘what drugs should I use?’ … I think sometimes nurses take on so much that they think ‘I have to know what drug to give, how much to give, where to give it, when to give it’ (RVN 5).

These experienced RVN interviewees emphasised the collaborative nature of the work and spoke about the need to clarify for newly qualified veterinary nurses the limit of their responsibilities within the clinical care team. While it was important that RVNs recognised problems as soon as they arose and flagged them, it was then up to their colleagues to work together to resolve the situation. “So especially for new grads if they’re monitoring on their own. They’re worried ‘what if the blood pressure drops and I have to get this drug?’ And I’m like ‘no, you just have to have that on hand’. The vet then like [sic] jumps in” (RVN 6).

Mentoring challenges included time pressures as well as receptiveness of recent graduates, with one participant expressing a desire for further training to better manage such problems.

Maybe be a bit humble about it. The fact that just because you have done [3-4] years [in college] and qualified, you're not experienced … recently we had advertised a position for a qualified nurse and hired a qualified nurse. She was qualified 18 months and it just wasn't there you know. There was a bit of an issue where it felt like because she was qualified, she felt like she did know it, but she didn't (RVN 4).

While this participant spoke of their interest in additional training, none of the RVNs reported receiving any formal support in mentoring newly qualified colleagues.

## Discussion

### Overview

The RVNs and veterinary nursing students who contributed to this study differed in their views of what a competent veterinary nurse can do, how long it takes to become competent and the level of clinical experience in the workplace needed to attain it. Students saw competence primarily as the combination of theoretical knowledge with psychomotor skills to enable the provision of clinical patient care. They acknowledged confidence and professional behaviours as also being involved but put less emphasis on their scope and importance than did RVNs. RVNs expected a competent RVN to provide leadership within the practice team. Students expected to be competent soon after graduation but RVNs felt it would take two years on average to develop. Students viewed workplace experience as a separate entity to competence. RVNs, in contrast, felt that the two were inherently linked, with experience being necessary for a veterinary nurse to become competent.

### The need for clear explanations of ‘competence’

These findings are of relevance to veterinary nursing educators as they indicate the practical importance of clear explanations of terms such as ‘day one competence’ and a ‘competent veterinary nurse’. Previous reports in the nursing literature have highlighted the challenges such variation in competence expectations poses for both students and their supervisors [28, 31, 54]. To address this problem, educators should explicitly inform both students and work placement supervisors what it is that we expect a newly qualified veterinary nurse to be able to do and (arguably more importantly) not do. Both new graduates and their colleagues/employers should be clear that a period of clinical experience in the workplace is needed to transform ‘day one competence’ into ‘professional’ or independent competence. In addition, the support that recently qualified RVNs receive is critical to the successful attainment of this mutually beneficial goal.

### RVN expectations of a competent veterinary nurse

The components of competence identified by the participants in this study (knowledge, skills and attributes of professional behaviour), correspond to those identified in the European framework for the profession [1], as well as previous reports on medical and nursing competence [10, 11, 27]. The importance placed by RVNs on workplace experience in the attainment of competence mirrors the views previously reported by experienced nurses in the field of medical nursing [4].

Leadership is identified as a component of competence in some nursing competency frameworks [55–58]. A recent study of 299 Australian nurses found that only 12.7% expected a newly qualified nurse to be competent in this skill area [31]. The Dossier of European Competencies for the Veterinary Nurse includes the establishment and maintenance of working relationships as a competency [1]. However, while the ability to assume a clinical leadership role when appropriate is referred to as a day one skill by the RCVS [2], it is not mentioned by either ACOVENE or the Veterinary Council of Ireland (VCI) [1, 59]. Leadership may be a component of ‘professional’ rather than ‘day one’ veterinary nursing competence and further research to explore the contribution of veterinary nurses to the management of practice teams would be beneficial.

This study adds the perceptions of members of the veterinary nursing profession to the literature on competence. The views of the participants in this study mirror those reported in the nursing literature. Nursing students have reported a similar focus on clinical proficiency as the main constituent of competence [60], whilst their practicing colleagues felt that competence encompasses a broader range of skills and attributes [61].

This study found that RVNs linked confidence with competence. This linkage has also been reported in the nursing literature [60, 62], where confidence is seen as necessary to translate knowledge into clinical practice. However, the exact relationship between these two concepts remains unclear [60, 63]. The Conscious Competence Model, which has been attributed to Noel Burch [64], describes individuals moving from unconsciously incompetent via conscious incompetence and conscious competence to unconscious competence. This model’s third stage (conscious competence) is associated with learner confidence in their ability to complete a task, coupled with an awareness of their limitations [65, 66]. The results of this study are a preliminary indication that confidence is also perceived as being related to veterinary nursing competence. This topic warrants further exploration, as efforts to develop or improve learner confidence may facilitate the development of competence.

It was interesting that RVNs in this study described a lack of role clarity amongst recent veterinary nursing graduates. They reported a tendency for recently qualified veterinary nurses to assume responsibility for some aspects of patient care that were beyond their remit. These RVNs recognised this as a potential source of anxiety for newly qualified veterinary nurses, mirroring Benner’s observation that the transition to the workplace is often associated with significant anxiety amongst nurses [19]. A UK study also reported high anxiety levels amongst undergraduate veterinary students, due to the students placing unrealistically high expectations of competence on themselves at the point of graduation [35].

The RVNs in this study reported that they helped recent graduates to correctly focus on their patient care responsibilities. However, recent veterinary nursing graduates may find themselves employed as the sole RVN in a practice, or experienced colleagues may not always be available for guidance. This study identified a potential need for educators and regulators to ensure students can identify their patient care responsibilities accurately as they enter the workplace, to reduce both their anxiety levels and the risk of patient harm if attempting to take on too much. One RVN participant indicated a desire for mentorship training and this is an area that could be examined by continuing veterinary education (CVE) providers.

### Study limitations

Weaknesses of this study include the small numbers of participants and the use of convenience sampling. The students were in the first year of their studies, as the aim was to examine what perceptions they held at the onset of training. However, their views may change as they progress through the course. In addition, the survey response rate amongst RVNs was low, at 14%. The RVN survey link was shared as widely as possible via the IVNA and the DkIT practice placement database. Lack of access to email addresses for an entire research population may reduce response rates [49] and the British Veterinary Association have noted that high workloads in veterinary practices may reduce the time available to engage with research data collection [67]. Previous online surveys of Irish veterinary professionals have reported response rates of between 3.5 and 44% [53, 68, 69].

Concerns exist that differences between respondents and non-respondents may introduce bias [45, 70]. However, a study of survey response rates in nursing research found few differences between nurses that did and did not participate [71]. The gender, average age, employment type and educational background of the RVN survey respondents resembled those reported for the profession overall [53]. All the interviewees had direct experience of student supervision, so their views on competence may not be representative of Irish RVNs in general.

Low response rates and small sample sizes reduce the generalisability of the results. However, it has been pointed out that small-scale surveys using non-probability sampling retain their value in social research studies where the intention is to produce an exploratory sample, rather than a representative one [49]. From this pragmatic perspective this study provides an initial overview of competence perceptions in this previously unexamined area and has provided direction for future competence studies.

### Future research

Further studies with larger numbers of participants, plus employers, could establish the repeatability of these findings. An evaluation of the ability of both veterinary nursing students and RVNs to select the same descriptors that describe competence could be informative. A study of veterinary nursing students at the point of graduation which examined how their perceptions of competence change as they train would also be of interest, as would further work to establish the patient care responsibilities newly qualified veterinary nurses expect to take on and the role of learner confidence in competence development.

## Conclusions

This study contributes to the literature by providing preliminary evidence of the views of competence held by veterinary nursing students and RVNs. The RVNs and students perceived competence in different ways. This agrees with previously reported findings in the nursing literature. Students and new graduates are focused on clinical skills, whilst RVNs perceive this is to be just one, albeit important, aspect of a broader definition of competent performance.

Educators and regulators should explicitly state what precisely they mean when using terms such as ‘competent’ or ‘day one competencies’. This would help to ensure that all stakeholders are clear and in agreement as to what constitutes competent performance by a veterinary nurse, both at graduation and as their career progresses.

## Supplementary information

**Additional file 1.** Veterinary nursing competence survey

## Data Availability

The datasets generated and analysed during this study are available from the corresponding author on reasonable request.

## Abbreviations

ACOVENE: Accreditation Committee for Veterinary Nurse Education
CVE: Continuing veterinary education
DkIT: Dundalk Institute of Technology
IVNA: Irish Veterinary Nursing Association
RCVS: Royal College of Veterinary Surgeons
RQ: Research question
RVN: Registered Veterinary Nurse
UCD: University College Dublin
VCI: Veterinary Council of Ireland
