# Supplementary material for: Competence perceptions of veterinary nursing students and registered veterinary nurses in Ireland: a mixed methods explanatory study
Source: Ir Vet J. 2020 Jun 17;73:10. doi: 10.1186/s13620-020-00162-2 (PMC7301512; doi:10.1186/s13620-020-00162-2)
Supplement: Supplementary file 1 — Additional file 1. Veterinary nursing competence survey [file 13620_2020_162_MOESM1_ESM.pdf]

Veterinary nursing competence survey

1. In what year were you born? (enter 4-digit birth year; for example 1992)

2. What is your gender?

O Female

O Male

3. *What is your veterinary nursing qualification?

O UCD diploma (level 7)

O UCD degree (level 8)

O DkIT degree (level 7)

O LyIT degree (level 7)

O AIT degree (level 7)

O St John’s Diploma (level 6)

O Other (please specify)

4. *In what year did you qualify as a veterinary nurse? (enter 4-digit year; for example 2012)

5. *Please indicate the type of work you are currently involved in.

O Companion animal practice

O Mixed practice

O Equine practice

O Farm animal practice

O Exotics

O Other (please specify)

6. What does the word “competent” mean to you?

7. When do you think a veterinary nurse becomes competent?

O During college

O By graduation

O In the 1st year after graduation

O 4-5 years after graduation

O > 5 years after graduation

O >10 years after graduation

O 2-3 years after graduation O I don’t know

Comment (optional)

8. When do you think a veterinary nurse becomes experienced?

O During college

O 4-5 years after graduation

O By graduation

O more than 5 years after graduation

O In the 1st year after graduation

O more than 10 years after graduation

O 2-3 years after graduation

O I don’t know

Comment (optional)

* RVN survey only
